# Supplementary material for: MsYSL6, A Metal Transporter Gene of Alfalfa, Increases Iron Accumulation and Benefits Cadmium Resistance
Source: Plants (Basel). 2023 Oct 5;12(19):3485. doi: 10.3390/plants12193485 (PMC10575464; doi:10.3390/plants12193485)
Supplement: Supplementary file 1 [file plants-12-03485-s001.zip › plants-2624615-supplementary.pdf]

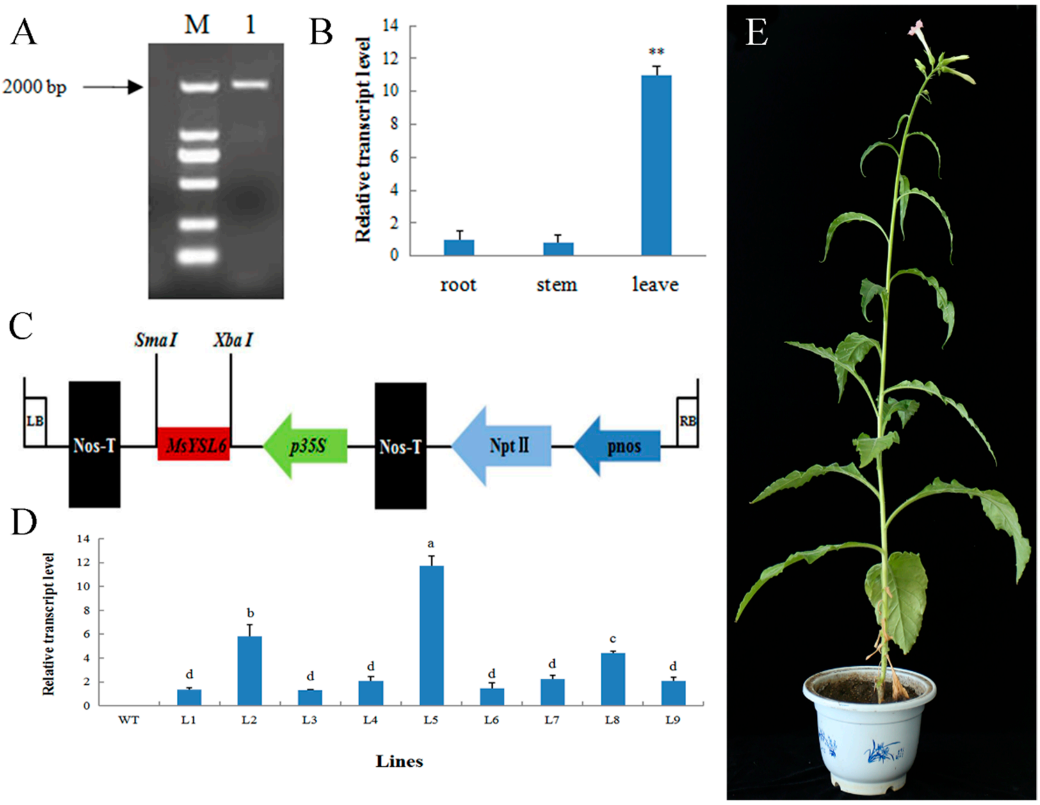

Figure S1. The cloning of *MsYSL6* from alfalfa and the transformation into tobacco. (A) The full-length of *MsYSL6* amplified by PCR from complementary DNA (cDNA) of alfalfa. M: DL 2000; 1: PCR product (*MsYSL6* CDS with 2, 028 bp). (B) Construction of pBI121-*MsYSL6* vector. LB: T-DNA left border; RB: T-DNA right border; p35S: cauliflower mosaic *virus* promoter; NptⅡ: Neomycin phosphotransferaseⅡ gene. (C) The transcript levels of *MsYSL6* in transgenic tobacco. Different letters represent significant differences (p <0.05). (D) The *MsYSL6* transgenic tobacco plant.

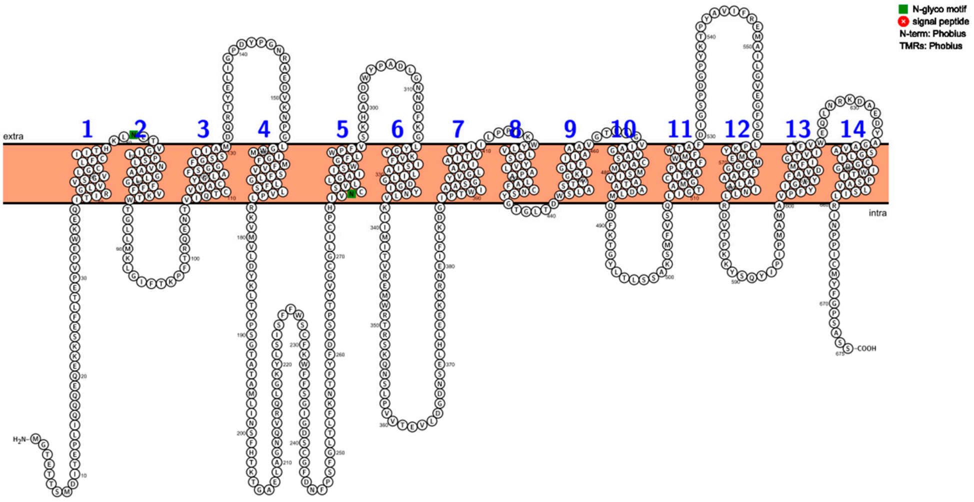

Figure S2. The predicted transmembrane domains of MsYSL6 protein.

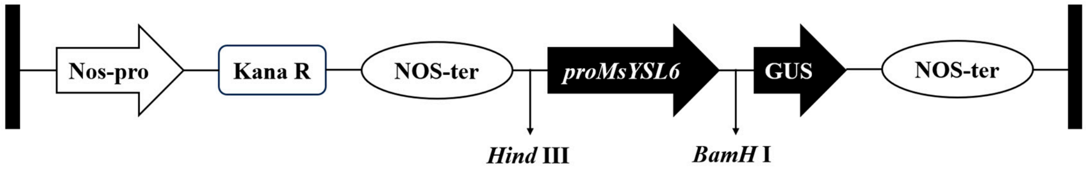

Figure S3. Construction of pBI121-*MsYSL6*pro::GUS.

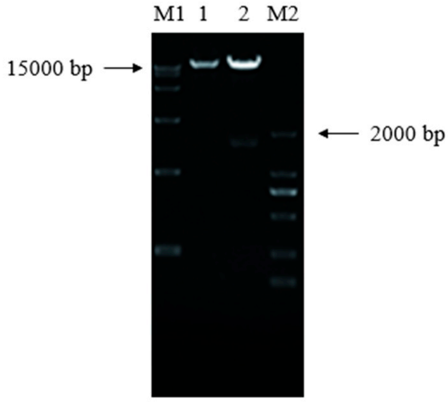

Figure S4. Identification of pBI121-*MsYSL6*pro::GUS. M1, Marker DL15000; M2, Marker DL2000; 1, pBI121-pro*MsYSL6*::GUS; 2, Recombinant plasmid digested by BamH I, Hind III.

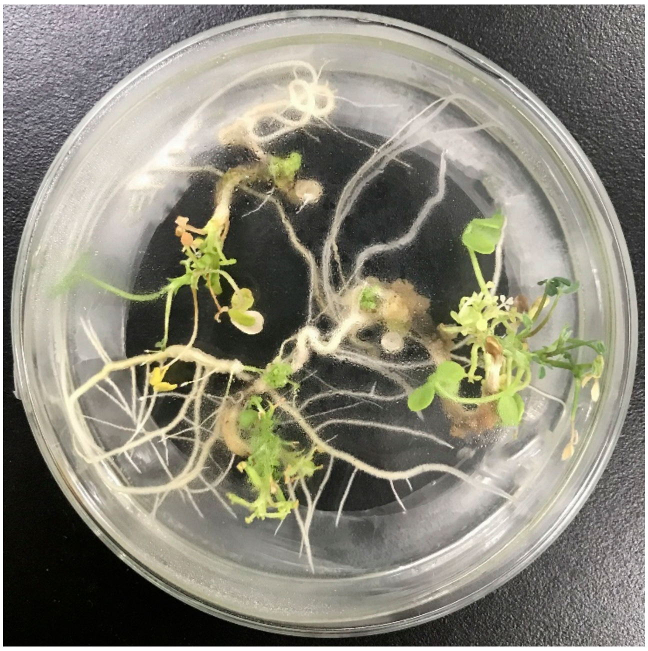

Figure S5. The pBI121-*MsYSL6*pro::GUS induced alfalfa hairy roots.

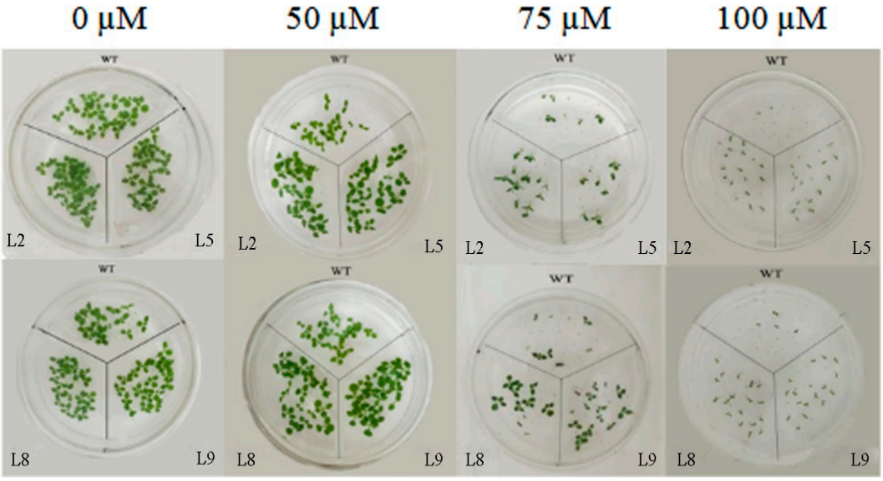

Figure S6. The germination of *MsYSL6*OE tobacco under Cd stress. The seeds of four *MsYSL6*OE lines (L2, L5, L8, and L9) and WT tobacco were germinated on a half-strength MS solid medium containing 0, 50, 75, 100 μM $CdCl_2$.

Table S1. The germination rates of *MsYSL6Ex* lines and WT under Cd stress.

Line	Cd			
	Control	50 μM	75 μM	100 μM
WT	95.00±0.05	75.00±0.05	45.00±0.05	26.67±0.06
L2	96.67±0.03	83.33±0.03*	66.67±0.03*	53.33±0.03*
L5	95.00±0.05	83.33±0.03*	68.33±0.03*	55.00±0.00*
L8	96.67±0.03	83.33±0.03*	68.33±0.03*	55.00±0.05*
L9	96.67±0.03	83.33±0.03*	66.67±0.03*	50.00±0.05*

Note: *$p < 0.05$.

Table S2. The primer sequence.

Primer Comments	Primer Sequence
MsYSL6 cloning	F: ATGGGTACAGAAACA<br>R: TCAGCTGCTTGCGGAA
qRT-PCR of *MsYSL6*	F: CTCAGTCACAACGGGAAGG<br>R: TGGAACAGCCACAGCAAT
*MsYSL6*pro fragment cloning	F: CGGGATCCGCTATCTATTATGTGCTATCT<br>R: CCAAGCTTGTTTGAGGTGAATCTGAG
MsNAS gene cloning	F: CGGGATCCTATAGAGATGATACCCACCCG<br>R: GAAGATCTTGGATTGAATTGAAGTAATTTTA

qRT-PCR of *MsNAS*	F: AAGATTGTGGCTTCGGAT<br>R: TTCACTCCTACGCATACCA
*MsNAS*pro fragment cloning	F: GGGATCC TATAGAGATGATACCCACCCG<br>R: GAAGATCTTGGATTGAATTGAAGTAATTTTA
MsACTIN	F: ACGAGCGTTTCAGATG<br>R: ACCTCCGATCCAGACA
NtGAPDH	F: TAAGGGTGGTGCCAAGAAGGT<br>R: AGCAAGAGGAGCAAGGCACTT
